# Socio-Demographic Determinants of Dietary Strategies of Mothers of School-Aged Children—A Study in Pomeranian Province

**DOI:** 10.3390/nu17223514

**Published:** 2025-11-10

**Authors:** Łukasz Długoński, Magdalena Skotnicka, Anna Mikulec

**Affiliations:** 1 Department of Commodity Science, Faculty of Health Sciences, Medical University of Gdansk, 7 Debinki Street, 80-210 Gdansk, Poland; dlugonski@gmail.com; 2 Faculty of Engineering Sciences, University of Applied Sciences in Nowy Sacz, 1a Zamenhofa Street, 33-300 Nowy Sacz, Poland; amikulec@ans-ns.edu.pl

**Keywords:** feeding practices, nutritional parenting strategies, child nutrition, CFPQ

## Abstract

Background: Parents’ dietary strategies shape children’s eating habits. This study investigated socio-demographic determinants of maternal feeding practices among school-aged children in the Pomeranian province of Poland. Using a cross-sectional survey conducted in July 2025, we compared feeding strategies based on family structure, maternal employment, and number of children, and identified distinct parenting profiles through cluster analysis. Methods: A cross-sectional survey was conducted in July 2025 among 719 mothers of elementary school children in Pomeranian Voivodeship, using a convenience sampling design. An abbreviated version of the Comprehensive Feeding Practices Questionnaire (CFPQ) with 16 items across eight subscales was used. ANOVA compared feeding strategies between groups, Spearman correlations examined associations, and k-means cluster analysis identified maternal parenting profiles. Results: Encouragement and modeling were the most frequent strategies, while monitoring was least common. Mothers raising children with a partner and those employed used monitoring, modeling, and encouragement more often. Single or non-working mothers relied more on food as a reward and for emotion regulation. Mothers of only children applied control and monitoring less intensively than mothers with multiple children. All strategies were positively correlated. Cluster analysis identified three parenting profiles: intensely directive, moderate, and emotional-supportive. Conclusions: Maternal feeding strategies vary with socio-demographic factors. Educational interventions promoting healthy eating should be tailored to family structure and mothers’ employment status.

## 1. Introduction

Since the beginning of the 21st century, the health of school-age children has been recognized as one of the key public health priorities worldwide [1,2,3]. The physical and mental well-being of children, who are in a period of intensive development, is important not only for their individual functioning but also for the future human capital of society. Over the past two decades, children have increasingly struggled with various health problems, such as deteriorating eyesight, excessive weight, and mental health issues [4]. In Poland, despite alarming rates of overweight and obesity among children [5,6], there is still a lack of research comprehensively analyzing the socio-demographic determinants of mothers’ feeding strategies. In particular, the interaction between family structure, the mother’s occupational status, and the number of children in the household has not yet been thoroughly investigated in this region. Health problems at school age are associated with negative consequences throughout life, increasing the risk of serious complications such as cardiovascular disease and type 2 diabetes [7]. Longitudinal data from the Bogalusa Heart Study indicate that risk factors such as obesity and hypertension in children often persist into adulthood [8]. In addition, these diseases generate increasing treatment costs and affect children’s social functioning through school absenteeism, educational losses, and reduced future productivity [9].

The early development of healthy eating habits is crucial, as it determines energy balance, current body weight, and its changes in the long term [10,11]. Making the right dietary choices affects not only physical health but also the mental well-being and overall well-being of children [10]. Parents play a special role in this process, as they create the eating environment that shapes taste preferences and attitudes towards food [12,13]. How mothers choose food products, what verbal and non-verbal messages they convey during meals, and how they regulate access to food have a significant impact on children’s behavior. Strategies of excessive control and pressure to eat can lead to overeating or selective eating, while positive patterns, such as modeling or supporting the child, promote the maintenance of a healthy body weight [14,15]. The Comprehensive Feeding Practices Questionnaire (CFPQ) is used to comprehensively assess parenting practices. It allows for the analysis of many dimensions of behavior and has been used in various cultural contexts [16]. There are many tools designed to measure eating habits, preferences, and behaviors in children and parents (Child Feeding Questionnaire—CFQ or Parental Feeding Style Questionnaire—PFSQ) [17,18]. However, from the perspective of the proposed strategies, the CFPQ is particularly interesting, as it captures a broad spectrum of feeding practices and enables the identification of both supportive and potentially harmful approaches. The CFPQ subscales reflect different parenting styles. Encouragement and modeling are closer to a supportive approach, while control and food rewards are more associated with a restrictive style. This allows the tool to assess not only feeding practices but also parents’ broader approach to parenting.

The choice of feeding strategies is influenced by a wide range of factors: parents’ level of education, financial situation, cultural norms, food availability, and general living conditions [19]. Although these factors are important and well-documented in the literature, not all of them can be captured in population-level studies. Therefore, this study focuses on three key socio-demographic determinants that directly shape the organization of family life and parenting decisions: family structure (single motherhood vs. co-parenting), maternal employment status (employed vs. not employed), and the number of children in the household. Research suggests that working mothers are more likely to use monitoring and modeling strategies, while not being employed encourages more frequent use of food as a reward or to regulate emotions [20]. Similarly, having more children may be associated with less control over diet and difficulties in monitoring consumption [21]. Although numerous studies have analyzed feeding practices in Western countries, there is still limited research examining how these three factors interact in Central and Eastern European contexts, including Poland. Although parental feeding practices have been the subject of numerous studies in Western countries [22], the context of Central and Eastern Europe, including Poland, remains relatively poorly understood. Studies conducted in countries such as Croatia [23] emphasize the importance of cultural and social factors in shaping feeding practices.

Filling this research gap is crucial for designing effective and context-specific interventions that promote healthy eating habits among children. Therefore, the aim of this study was to comprehensively analyze the feeding practices of mothers of school-age children in the Pomeranian Province, with particular emphasis on their links to three socio-demographic factors.

## 2. Materials and Methods

### 2.1. Study Design

A questionnaire based on the Comprehensive Feeding Practices Questionnaire (CFPQ) was used in this study. It consisted of 16 single-choice questions. The questionnaires were completed online by mothers of children attending elementary schools in Pomeranian Voivodeship, Poland, in July 2025. Participants were recruited using a convenience sampling approach. This study employed a cross-sectional design, collecting data at a single time point. To ensure response consistency, participants were provided with the following instruction: Please answer all the following questions with regard to one specific child in your household. If you have more than one child, please refer to the oldest child who currently attends elementary school. Data was collected using an anonymous online survey developed in Google Forms. The survey was distributed in cooperation with elementary schools, through those responsible for digital administration (e.g., secretariats, IT coordinators). The link was made available through official school websites, social media, and the Librus school communication system—a widely used electronic gradebook and communication system in Polish schools, providing access to parents, teachers, and administrators. Written consent to participate in the survey was included. The survey was fully anonymous, and the results were used for research purposes only. Participation in the survey was voluntary. The survey was conducted in accordance with ethical principles, including non-harm, beneficence, justice, and autonomy, in accordance with the Declaration of Helsinki (2000). Personal data was anonymized in accordance with the Regulation (EU) 2016/679—the General Data Protection Regulation (GDPR) [24]). The study was approved by the Bioethics Committee of the Medical University of Gdansk, Poland [KB/322/2025].

### 2.2. Comprehensive Feeding Practices Questionnaire

The Comprehensive Feeding Practices Questionnaire (CFPQ) was chosen for this study over other available instruments due to its multidimensional structure, which comprehensively assesses both positive and controlling feeding practices relevant to school-aged children. The Comprehensive Feeding Practices Questionnaire (CFPQ) is a tool developed by Musher-Eizenman and Holub (2007) [25] to assess a variety of parenting strategies related to child feeding. It originally included 49 items divided into 12 subscales: monitoring, emotion regulation, control, environment, food as a reward, teaching about nutrition, restriction for health, restriction for weight, child control, modeling, involvement, and encouraging balance and variety. Our study included 16 items representing 8 of these subscales. Selection was based on previous work which showed that some of the subscales have similar theoretical relevance or are less frequently used in research with parents. Among others, the subscales “teaching about nutrition,” “pressure,” “restriction for weight,” and “child involvement” were excluded due to their low level of relevance in the context of school-age children’s behavior or theoretical overlap with other scales (e.g., “restriction for weight” with “restriction for health” or “pressure” with “child control”). The goal was to create an abbreviated, as representative as possible version of the tool, containing 2 questions from each of the 8 key subscales. This approach has been previously used and validated in studies involving fathers and mothers, among others [26,27,28]. Table 1 includes the subscales with their corresponding questions.

A five-point Likert scale [29] was used to rate all items, where 1—never/strongly disagree, 2—rarely/partially disagree, 3—sometimes/neutral, 4—often/partially agree, and 5—very often/completely agree. Based on the responses to the CFPQ (Comprehensive Feeding Practices Questionnaire), total scores were calculated for each of the eight subscales:

I. Monitoring—the mother’s control of the amount and frequency of the child’s consumption of unhealthy foods (e.g., sweets, snacks).
II. Emotion Regulation—giving the child food in response to his emotions, such as boredom or sadness.
III. Control—adjusting meals according to the child’s preferences and having no restrictions on the type of food consumed.
IV. Environment—the availability of food products in the home, including snacks and sweets.
V. Food as a Reward—using food as a form of reward or punishment.
VI. Modeling—demonstration of healthy eating behaviors by the mother as a role model.
VII. Encourage—encouraging the child to prepare meals and try new foods.
VIII. Restriction for Health—limiting the consumption of unhealthy foods for the sake of the child’s health.

The total score for each of the eight subscales was calculated as the sum of the points from the two corresponding items, which means that the range of possible scores was from 2 to 10. Next, for the entire sample, the arithmetic means ($\bar{x}$) and standard deviations (SD) of these total scores were calculated. The values presented in the Results section therefore refer to the subscale sums and not to individual items in the questionnaire. The arithmetic mean ($\bar{x}$) and standard deviation (SD) were then calculated for each subscale to describe the overall level of mothers’ use of each parenting strategy.

### 2.3. Participants

The survey was conducted in July 2025 in elementary schools in Pomeranian Voivodeship, Poland. 732 mothers participated in the survey. Purposive sampling based on respondents’ availability was used. Participants in the survey were mothers of children attending elementary schools who responded to the questionnaire provided online. Distribution of the questionnaire was performed through the official websites and social media of the schools. This approach was intended to increase the representativeness of the results and allow analysis of the influence of various environmental factors on children’s eating attitudes. Participating in the study was voluntary. All mothers who provided informed consent were included, with no specific exclusion criteria applied, which allowed for a broad sample reflective of the school’s population. Ultimately, 719 women participated in the study after excluding incomplete responses (*n* = 13). This corresponds to a final response rate of 98.2%. Family status was determined by respondents’ self-report. Mothers raising a child with a partner were considered women who declared cohabitation and shared responsibility for the child with a partner or husband (including the child’s father). Single mothers were women who did not indicate any partner as a co-parent. The largest group was women aged 30–39 (57.86%), while the least numerous were women aged 40 and over (18.22%). The majority of female respondents declared to be professionally active, 60.08% (Table 2).

### 2.4. Statistical Analysis

The statistical analysis was performed using the PQStat Software (2023) v.1.8.6.102.

The internal consistency for the subscales, assessed using Cronbach’s alpha, ranged from 0.62 to 0.87, indicating acceptable to good reliability for the applied instrument in the study sample. The data were subjected to statistical analysis, calculating mean values for each of the eight strategies. For each question, a quantitative analysis and a chi-square test of fit were performed to assess whether the distribution of responses differed significantly from a uniform distribution. The *p*-value was interpreted with reference to the accepted level of *p* = 0.05. A one-way analysis of variance (ANOVA) was conducted to assess the effect of family and occupational status of mothers on the use of parenting strategies for child nutrition. To examine whether the number of children in the family differentiates the use of child-rearing strategies in the area of nutrition, eight one-way analyses of variance (ANOVAs) were conducted, with the number of children in the household as the grouping variable. In addition, a Tukey post hoc test will be performed, if necessary, to further identify the sources of differences between the different groups of parents. To assess the relationship between the various parenting strategies related to child nutrition, Spearman’s non-parametric rank correlation coefficient was used. The choice of this method was justified by the result of the Shapiro–Wilk test, which showed the lack of normality of the distribution of the studied variables and the ordinal nature of some of the data. In order to identify patterns of parenting strategies used by parents in the area of child nutrition, cluster analysis was carried out using the k-means clustering algorithm. The number of clusters was determined by the silhouette score, which indicated the optimal division into three groups. 

## 3. Result

### 3.1. General Characteristics of Maternal Feeding Strategies

The study evaluated eight strategies related to control and regulation of eating behavior: monitoring, emotion regulation, control, environment, food as a reward, encouragement, modeling, and restriction for health. 

As shown in Table 3, the strategies most frequently used by mothers were encouragement and modeling, which achieved the highest mean scores. In contrast, monitoring was the least frequently reported strategy among the studied feeding practices. This means that mothers most often used precisely these parenting strategies in the context of nutrition, that is, they used encouraging the child to try new foods and participate in preparing meals. On the other hand, modeling often presented positive nutrition patterns in the family, such as eating healthy foods in the presence of the child and talking about their benefits. In contrast, the lowest mean value was observed for monitoring ($\bar{x}$ 6.83), which may indicate that mothers are less likely to use strategies that involve active control over what and how often the child eats—especially with regard to products considered unhealthy, such as sweets and salty snacks. The results suggest that the surveyed group is most likely to base their strategies on encouraging and promoting appropriate eating behaviors, thus limiting the benefits of controlling and monitoring (Table 3).

### 3.2. Statistical Analysis of the Distribution of Responses Among Mothers Regarding Children’s Feeding Behavior

In the analysis of mothers’ eating behaviors toward their children, a number of important attitudes and habits were identified that may have a significant impact on the formation of children’s preferences and lifestyles. Based on the results obtained and the chi-square test (χ^2^) conducted, it was found that the distribution of responses for all 16 questions differed significantly from the random distribution (*p* < 0.05), confirming that the mothers surveyed present clear, well-established behavioral patterns.

Analysis of the mothers’ responses regarding their children’s eating behaviors revealed a range of ambivalent and varied strategies. The results from the monitoring subscale indicate that many mothers report being aware of the amount and frequency of their child’s consumption of sweets and salty snacks, suggesting active monitoring of these dietary aspects. At the same time, however, there is a noticeable tendency to use food as a tool for regulating emotions, both in the context of boredom and sadness—by offering food to the child regardless of perceived hunger. Among the mothers surveyed, the practice of giving in to the child’s preferences—such as preparing alternative meals when the child does not accept what has been served or allowing the child to freely choose food—is quite common. Analysis of responses revealed several key patterns. A majority of mothers reported frequently accommodating their child’s food preferences, with over half (58.6%) often or very often allowing the child to eat what they want. Furthermore, more than one-third of mothers frequently prepared an alternative meal if the child disliked what was served. These practices indicate a tendency to avoid conflict at mealtimes. Interestingly, despite this, a significant proportion of mothers also reported positive modeling behaviors, such as talking about the benefits of healthy food (Table 4). This indicates an attempt to reduce conflict while weakening the dietary structure. On the other hand, many mothers show positive modeling, try to eat healthy in the presence of the child, talk to the child about the virtues of healthy food, and actively encourage the consumption of a variety of products. It is also worth noting the presence of inconsistent control strategies. Some mothers admit that they reward their child with sweets for good behavior while restricting their access to them in response to undesirable behavior (Table 4). 

### 3.3. Comparison of Parenting Strategies for Child Feeding According to Family Status (Joint Parenting with Father/Partner vs. Single Parenting)

The purpose of the analysis was to examine whether the mother’s family status of joint parenting with a partner/father or single parenting differentiates the use of parenting strategies for child nutrition. The results of a one-way analysis of variance (ANOVA) showed statistically significant differences (*p* < 0.05) for four of the eight strategies studied. Mothers raising their children jointly with a partner/father had significantly higher scores for monitoring the child’s eating behavior, controlling eating, using food as a reward, and modeling proper eating habits. For the other strategies, no statistically significant differences were found between the groups. The results suggest that the presence of a second parent/guardian in the household may promote the use of more varied and active eating strategies, especially those requiring consistent monitoring and modeling of behavior (Table 5).

### 3.4. Comparison of Parenting Strategies According to Mother’s Occupational Status

The next part of the study analyzed the association between maternal occupational status and the use of child feeding strategies. The analysis revealed significant differences in the strategies used by employed and non-employed mothers. Significant differences were indicated in the use of most of the studied feeding strategies according to the mother’s occupational status. Statistically significant differences (*p* < 0.05) were observed for seven strategies: monitoring, regulation of emotions, environmental influence, use of food as a reward, encouragement, modeling, and health restrictions. Only for the control strategy was there no significant difference between groups (*p* = 0.123). Working mothers had significantly higher levels of monitoring their children’s eating behavior, were more likely to encourage certain behaviors, were more active in modeling proper eating habits, and used health restrictions. In contrast, non-working mothers were more likely to use food in regulating emotions and to use food as a reward. The results suggest that a mother’s occupational status may significantly affect the choice and frequency of use of different parenting strategies in raising children. These differences may be due to different patterns of time organization, access to resources, and dietary priorities depending on the work situation. The results of the survey indicate the need to consider parents’ professional contexts in child nutrition education programs (Table 6).

### 3.5. Differences in Parenting Strategies According to the Number of Children in the Family

The final component of the comparative analysis was to see if the number of children in the family differentiated the use of parenting strategies in the area of nutrition. For this purpose, eight one-way analyses of variance (ANOVAs) were conducted, with the number of children in the household as the grouping factor. Seven of the eight strategies analyzed showed statistically significant differences between parent groups (*p* < 0.05). Only the encourage strategy showed no significant differences (*p* > 0.05). To further identify the source of the differences, post hoc (Tukey) tests were conducted to determine between which groups there were significant differences. After post hoc analysis, for the control strategy, the test showed significant differences between parents with four or more children and parents of only children (*p* = 0.002) and between parents with two children and parents of only children (*p* = 0.000). There was also a significant difference between parents of only children and parents of three children (*p* = 0.0115). This means that parents of only children use the control strategy significantly less intensively than parents with more children. For the emotion regulation strategy, there is a significant difference between two children and one child (*p* = 0.0001) and between one and three children (*p* = 0.0017). This allows us to conclude that parents with two or three children are more likely to use the emotion regulation strategy than parents of only children. In this case, the other comparisons showed no statistically significant differences. For the food as a reward strategy, there is a significant difference between two children and one child (*p* = 0.0003) and between one and three children (*p* = 0.0078). Parents of two and three children are more likely to use food as a reward than parents of only children. Other comparisons by number of children in the household were not statistically significant. For the environment strategy, there was a significant difference between one and two children in the household (*p* = 0.0037), as well as between one and three children (*p* = 0.0303). Parents with two and three children are more likely to target environmental factors (e.g., food availability, environment) than parents of only children. For monitoring strategies, the Tukey test revealed significant differences in two cases: when parents have four or more children compared to one child (*p* = 0.0435) and when there are two children in the family compared to one child (*p* = 0.0033). The analysis shows that parents of only children are less likely to monitor their child’s eating behavior than parents with more children.

### 3.6. Relationships of Feeding Strategies 

The next stage of the analysis examined the co-occurrence of different child feeding strategies. Spearman’s coefficients were used to assess the relationships between specific strategies. This was justified due to the non-normal distribution of the variables and the ordinal nature of some of the data, confirmed by the Shapiro–Wilk test. The results indicate the existence of consistent patterns of parenting behavior, which can form the basis for further factor analyses or cluster analysis. Eight strategies, including monitoring, emotion regulation, control, environment, food as a reward, encouragement, modeling, and restriction for health, were analyzed to examine the co-occurrence of different feeding strategies. All variables were measured on numerical scales. The results showed that all strategies were significantly positively correlated with each other (*p* < 0.05), indicating a tendency for parents to use different parenting approaches together. The strongest correlations were noted between control vs. emotion regulation, control vs. environment, control vs. food as a reward, and environment vs. food as a reward. The values of the correlation coefficients suggest that strategies of a directing or regulating nature often co-occur. Parents who use control simultaneously monitor their child’s environment and use food as a reward tool (Table 7).

### 3.7. Identification of Patterns of Parents’ Feeding Strategies—K-Means Cluster Analysis

Finally, as a summary of the parenting strategies analyzed, an attempt was made to identify patterns of parenting strategies used based on k-means cluster analysis (K-means). The data were previously standardized. Three sets were proposed. Each of the identified sets was described on the basis of the mean values of the variables included in the analysis, which made it possible to identify three distinct parenting profiles:

Set 0—Moderate: average intensity of all strategies, no dominant approach—possible interpretation as balanced or adaptive parenting.
Set 1—Intensely Directing: high intensity in all strategies, especially controlling and supportive, suggesting a high-involvement and structure-based style.
Set 2—Emotional-supportive: high intensity in positive reinforcement strategies (encouragement, modeling), low use of control and regulation of emotions by eating.

Based on the silhouette factor, three profiles were identified as child-feeding parenting strategies.

Set 0—Moderate (n = 365).

The characteristics of this set are the average values of strategies oscillating around 6. Neither the control strategy nor the support strategy is dominant, which may indicate a balanced parenting strategy or a flexible approach with no clear dominance of one strategy.

Set 1—Intensely directing (*n* = 247).

Parents belonging to this set were characterized by high values in all analyzed strategies, in particular control (8.26), environment (8.33), emotion regulation (8.07), and monitoring (7.60). Also high in supportive strategies: encourage (8.16) and modeling (8.28). This profile may correspond to a parenting strategy with a high degree of involvement and control over the child’s environment and emotions.

Set 2—Emotional-supportive (*n* = 107).

Parents in this set use very high support and modeling: encouragement (8.79), modeling (8.41), low emotion regulation (4.18), and food as a reward (4.60). Medium monitoring and control. This may be indicative of a parenting strategy based on positive reinforcement and one’s own example, without relying on the tools of control or food as a reward.

**Figure 1:**
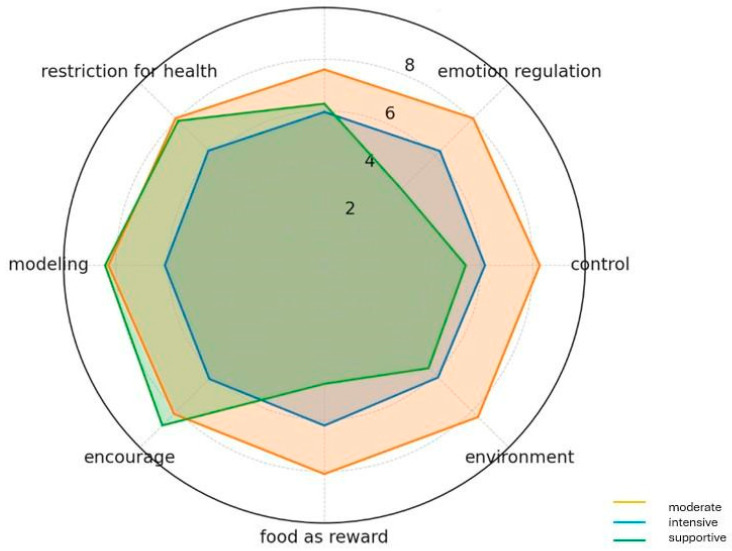
Profiles of parents’ eating strategies extracted using the K-means method.

The analysis revealed the presence of three distinct parenting profiles, varying in intensity and type of strategies used. These profiles are not merely statistical constructs but represent coherent and meaningful patterns of feeding practices. The ‘Intensely Directing’ profile reflects a highly structured, parent-centered approach, while the ‘Emotional-Supportive’ profile represents a more child-centered, autonomy-supportive style. The ‘Moderate’ group likely employs a flexible, context-dependent mix of strategies. The identified parenting strategies can provide a basis for developing more tailored educational and preventive programs for parents (Figure 1). Knowing that some parents use an intensely controlling approach, others a moderate approach, and still others an approach based primarily on support and modeling allows the content and methods of intervention to be tailored to the needs of each group. For instance, the ‘Intensely Directing’ group might benefit from guidance on fostering healthy child autonomy, while the ‘Emotional-Supportive’ group could be reinforced in their positive modeling behaviors. This makes it possible to more effectively support the formation of healthy eating habits for children.

## 4. Discussion

This study identified the feeding strategies most frequently used by mothers of school-aged children. Encouragement ($\bar{x}$ 7.60) and modeling ($\bar{x}$ 7.56) obtained the highest mean scores, suggesting that mothers in our sample favor supportive approaches to shaping their children’s eating habits. Similar results were reported by Vandenplas et al. and Mazza et al. [30]. From a practical perspective, these strategies should be emphasized in public health interventions, as they strengthen positive behaviors without coercion.

Monitoring was the least frequent strategy ($\bar{x}$ 6.83). Although less common, previous research indicates that even limited monitoring may positively influence children’s dietary patterns [31]. In our study, about one-third of mothers were aware of their children’s consumption of sweets and salty snacks. This finding aligns with previous studies using the CFPQ [32], suggesting that improving parental supervision could help reduce unhealthy snacking. These monitoring practices do not operate in isolation; parental strategies are often intertwined with emotional regulation and broader patterns of feeding behavior.

The strongest correlations were observed between emotion regulation and food as a reward and between control and emotion regulation. These findings are consistent with earlier studies showing that food-based emotion regulation is associated with weaker self-regulation in children [33,34]. Our results confirm that feeding strategies are not isolated but occur in patterns shaped by maternal emotional responses. Practically, this highlights the importance of supporting mothers in managing stress and emotions to prevent reliance on food as a tool for control or comfort.

The use of food as a reward emerged as a recurring theme. Mothers reported offering sweets when children were bored or sad, reflecting emotional feeding. Prior studies showed that these practices may increase caloric intake and promote emotional overeating [35,36,37]. Such mechanisms can impair children’s ability to self-regulate intake and should be discouraged. Public health programs should provide parents with alternative ways to reinforce positive behavior.

Children’s preferences also strongly influenced family practices. Some mothers allowed children to dictate food choices or prepared alternative meals when a dish was rejected. While this supports autonomy, it may encourage unhealthy eating. Greater availability of sweets and snacks at home, reported by many mothers, may further reduce diet quality [38]. On the other hand, involving children in cooking was common and aligns with previous findings that helping in the kitchen supports healthier food preferences [39]. These results suggest that interventions should encourage shared meal planning while maintaining clear family rules on food choices.

Sociodemographic factors were associated with feeding strategies. Mothers raising children with a partner reported greater use of modeling and monitoring. This finding aligns with the work of Douglas et al. (2024), who highlighted that the presence of a co-parent facilitates a more consistent and structured feeding environment, allowing for better monitoring and positive role modeling [40]. Working mothers scored higher across most supportive subscales (monitoring, encouragement, and modeling), a pattern also observed by Brito et al. (2024) in a large Brazilian cohort [41]. Mothers of only children reported less intensive use of control and monitoring compared to mothers with larger families. This is consistent with the findings of Hu et al. (2022), who suggested that as the number of children increases, parental resources and attention become diluted, which may lead to more frequent but less individualized control in response to household chaos [42]. These findings suggest that family structure and workload shape feeding practices and should be considered when designing interventions. Beyond individual sociodemographic influences, these feeding behaviors tend to cluster into distinct patterns, which can help identify groups of mothers who may benefit from targeted interventions.

Cluster analysis distinguished three profiles of feeding strategies, comparable to those described in adolescent populations [43]. These profiles have practical implications: “intensely directive” mothers may need guidance on balancing control with autonomy, the “moderate” group could benefit from reinforcement of positive strategies, while the “emotional-supportive” group should be encouraged to avoid permissive feeding. Tailoring educational programs to these profiles could improve their effectiveness.

The use of the validated CFPQ allowed a comprehensive assessment of diverse feeding practices. The relatively large sample adds value to our findings. However, several limitations must be acknowledged. First, the study relied on mothers’ self-reports, which may be affected by recall and social desirability bias. Second, the convenience of online samples limits representativeness, particularly regarding fathers and underrepresented groups. Third, no objective measures of children’s dietary intake were collected. Finally, the cross-sectional design prevents causal inferences between sociodemographic factors and maternal feeding practices. Additionally, the lack of exclusion criteria means that the sample may have included children with chronic diseases, medical dietary restrictions, or unusual care contexts. Such cases require specific nutritional strategies and may have introduced bias in comparisons between groups, limiting the interpretation of the results. Future studies should use longitudinal and observational methods, include more diverse samples, and consider variables such as income, education, and cultural background.

Our findings suggest that maternal feeding strategies are shaped both by personal factors (emotional regulation, responsiveness to children) and by family context (partnership status, employment, number of children). Public health programs should therefore address emotional aspects of caregiving, promote supportive strategies such as modeling and shared cooking, and discourage reward-based or permissive feeding. Tailoring interventions to parental profiles and family contexts may enhance their impact on the development of healthy eating habits in children. Based on the identified profiles, more targeted actions can be identified. For mothers who use an intensely directive approach, interventions that teach how to balance control with developing the child’s autonomy (e.g., psychological and nutritional workshops) may be beneficial. The emotionally supportive group, on the other hand, could be supported in maintaining positive modeling and encouragement through school programs or campaigns promoting joint meal preparation. The moderate profile, on the other hand, mainly requires reinforcement of existing practices, e.g., through simple educational materials and counseling in primary health care.

## 5. Conclusions

The study found that mothers most often use encouragement and modeling strategies when feeding their children, while monitoring is less common. Mothers raising children with a partner and those who are economically active tend to employ a broader range of strategies, including monitoring and health restrictions, whereas single or non-working mothers rely more on food to reward or manage emotions. The number of children also affects practices, with mothers of only children less likely to monitor, control, or reward with food. The use of different strategies was positively correlated, and cluster analysis identified three profiles of feeding practices: intensely controlling, moderate, and emotionally supportive, with the moderate profile being the most prevalent. These findings highlight the influence of socio-demographic factors on parenting strategies and suggest that nutrition education programs should consider family structure and parental employment to better promote healthy eating habits. Future research could explore the long-term impact of these strategies on children’s dietary behavior.

## Figures and Tables

**Table 1:** Comprehensive Feeding Practices Questionnaire.

Subscale	Question	Question Content
Monitoring	Question 1	Are you aware of how much and how often your child eats sweet things (e.g., candy, ice cream, cookies)?
	Question 2	Are you aware of how much and how often your child eats salty snacks?
Emotion Regulation	Question 3	Do you give your child something to eat or drink when he is bored, even if you think he is not hungry?
	Question 4	Do you give your child something to eat or drink when he is sad, even if you think he is not hungry?
Control	Question 5	Do you let your child eat what your child wants?
	Question 6	If my child doesn’t like what is served, I make my child something else.
Environment	Question 7	I usually have a lot of sweets at home.
	Question 8	I usually have a lot of snacks at home.
Food as a Reward	Question 9	I deny my child sweets in response to bad behavior.
	Question 10	I offer my child sweet things (e.g., candy, ice cream, cookies) as a reward when they have been good.
Modeling	Question 11	I tell my child that healthy food tastes good.
	Question 12	I try to eat healthy in front of my child.
Encourage	Question 13	I encourage my child to prepare family meals.
	Question 14	I encourage my child to eat a variety of foods.
Restriction for Health	Question 15	If I didn’t direct or regulate my child’s eating, my child would eat too much of my child’s favorite foods.
	Question 16	If I did not direct or regulate my child’s eating, he would eat too much junk food.

**Table 2:** Socio-demographic characteristics of the surveyed mothers for *n* = 719.

Variable	Category	*n*	%
Age	21–29 years	172	23.92
	30–39 years	416	57.86
	40 years and over	131	18.22
Occupational status	Works	432	60.08
	Does not work	287	39.92
Family status	Raises child/children with partner/husband	568	78.99
	Raises child/children alone	151	21.01
Number of children in the household	One child	358	49.79
	Two children	299	41.59
	Three children	49	6.81
	Four or more	13	1.81

*n*, number of mothers; %, percentage of mothers.

**Table 3:** Mothers’ nutrition strategies.

Nutrition Strategy	$\bar{x}$	SD
Monitoring	6.83	1.81
Emotion regulation	6.87	2.05
Control	7.12	1.79
Environment	7.18	1.85
Food as a reward	6.94	1.95
Modeling	7.56	1.70
Encourage	7.60	1.70
Restriction for health	7.43	1.78

$\bar{x}$, mean; SD, standard deviation.

**Table 4:** Distribution of mothers’ responses to questions about their children’s eating behavior.

No.	Question	Answers	*n*	%
**1**	Are you aware of how much and how often your child eats sweet things (e.g., candy, ice cream, cookies)?	never	30	4.17
		rarely	94	13.07
		sometimes	274	38.11
		often	243	33.80
		very often	78	10.85
**2**	Do you realize how much and how often your child eats salty snacks?	never	28	3.89
		rarely	81	11.27
		sometimes	202	28.09
		often	289	40.19
		very often	119	16.55
**3**	Do you give your child something to eat or drink when your child is bored even if you think your child is not hungry?	never	55	7.65
		rarely	70	9.74
		sometimes	198	27.54
		often	290	40.33
		very often	106	14.74
**4**	Do you give your child something to eat or drink when your child is sad even if you think your child is not hungry?	never	70	9.74
		rarely	73	10.15
		sometimes	192	26.70
		often	251	34.91
		very often	133	18.50
**5**	Do you let your child eat what your child feels like eating?	never	27	3.76
		rarely	55	7.65
		sometimes	216	30.04
		often	282	39.22
		very often	139	19.33
**6**	If my child does not like what is served, I make my child something else.	never	42	5.84
		rarely	99	13.77
		sometimes	188	26.15
		often	245	34.08
		very often	145	20.17
**7**	I usually have a lot of sweets at home.	disagree	48	6.68
		partially disagree	61	8.48
		neutral	154	21.42
		partially agree	317	44.09
		completely agree	139	19.33
**8**	I usually have a lot of snacks at home.	disagree	32	4.45
		partially disagree	74	10.29
		neutral	186	25.87
		partially agree	302	42.00
		completely agree	125	17.39
**9**	I deny my child sweets in response to bad behavior.	disagree	53	7.37
		partially disagree	74	10.29
		neutral	181	25.17
		partially agree	300	41.72
		totally agree	111	15.44
**10**	I offer my child sweet things (e.g., candy, ice cream, cookies) as a reward when my child is good.	disagree	64	8.90
		partially disagree	86	11.96
		neutral	158	21.97
		partially agree	277	38.53
		completely agree	134	18.64
**11**	I tell my child that healthy food tastes good.	disagree	18	2.50
		partially disagree	73	10.15
		neutral	156	21.70
		partially agree	295	41.03
		completely agree	177	24.62
**12**	I encourage my child to prepare family meals.	disagree	24	3.34
		partially disagree	79	10.99
		neutral	146	20.31
		partially agree	289	40.19
		totally agree	181	25.17
**13**	I encourage my child to eat a variety of foods.	disagree	12	1.67
		partially disagree	64	8.90
		neutral	132	18.36
		partially agree	311	43.25
		totally agree	200	27.82
**14**	I try to eat healthy with my child.	disagree	26	3.62
		partially disagree	73	10.15
		neutral	101	14.05
		partially agree	333	46.31
		totally agree	186	25.87
**15**	If I did not direct or regulate my child’s eating, my child would eat too much of my child’s favorite foods.	disagree	25	3.48
		partially disagree	80	11.13
		neutral	122	16.97
		partially agree	336	46.73
		completely agree	156	21.70
**16**	If I did not direct or regulate my child’s food, my child would eat too much junk food.	disagree	33	4.59
		partially disagree	58	8.07
		neutral	174	24.20
		partially agree	271	37.69
		totally agree	183	25.45

*n*, number of mothers; %, percentage of mothers.

**Table 5:** Comparison of scores on subscales of parenting strategies between mothers raising them with a partner versus alone.

Subscale	With a Partner		Alone		F	*p*
	$\bar{x}$	SD	$\bar{x}$	SD		
Monitoring	6.98	1.77	6.27	1.82	4.32	0.041 *
Emotion regulation	6.95	1.77	6.56	1.90	1.56	0.214
Control	7.25	1.75	6.62	1.86	5.87	0.018 *
Environment	7.33	1.82	6.64	1.87	0.87	0.354
Food as a reward	7.02	1.98	6.64	1.76	6.41	0.013 *
Encourage	7.74	1.64	7.06	1.81	1.23	0.271
Modeling	7.74	1.62	6.87	1.84	3.98	0.049 *
restriction for health	7.53	1.72	7.09	1.96	2.15	0.146

$\bar{x}$—mean; SD—standard deviation; * *p* < 0.05; F, ANOVA.

**Table 6:** Comparison of scores on subscales of parenting strategies between working and non-working mothers.

Subscale	Works		Does Not Work		F	*p*
	$\bar{x}$	SD	$\bar{x}$	SD		
Monitoring	6.98	1.73	6.62	1.90	11.23	0.000 *
Emotion regulation	6.78	2.17	7.01	1.84	5.67	0.000 *
Control	7.15	1.74	7.07	1.86	8.45	0.123
Environment	7.11	1.95	7.30	1.70	10.12	0.000 *
Food as a reward	6.84	2.11	7.08	1.67	15.89	0.000 *
Encourage	7.71	1.80	7.43	1.52	4.32	0.000 *
Modeling	7.67	1.77	7.39	1.59	6.78	0.000 *
Restriction for health	7.51	1.90	7.31	1.60	9.56	0.000 *

$\bar{x}$—mean; SD—standard deviation; * *p* < 0.05; F, ANOVA.

**Table 7:** Spearman correlation matrix between mothers’ eating strategies.

	Monitoring	Emotion Regulation	Control	Environment	Food as a Reward	Encourage	Modeling	Restriction for Health
Monitoring	x	0.346	0.398	0.389	0.268	0.180	0.224	0.185
Emotion regulation	0.346	x	0.585	0.457	0.494	0.130	0.224	0.220
Control	0.398	0.585	x	0.523	0.525	0.227	0.298	0.276
Environment	0.389	0.457	0.525	x	0.557	0.266	0.308	0.312
Food as a reward	0.268	0.494	0.525	0.557	x	0.250	0.320	0.291
Encourage	0.180	0.130	0.237	0.266	0.250	x	0.543	0.366
Modeling	0.224	0.224	0.298	0.308	0.320	0.543	x	0.388
Restriction for health	0.185	0.221	0.276	0.312	0.291	0.366	0.388	x

Spearman correlation test (r); *n* = 719; x indicates omitted diagonal values (constructs correlated with themselves).

## Data Availability

The original contributions presented in this study are included in the article. Further inquiries can be directed to the corresponding author.
